# Decolonisation, Indigenous health research and Indigenous authorship: sharing our teams’ principles and practices

**DOI:** 10.5694/mja2.52509

**Published:** 2024-11-11

**Authors:** Pat Dudgeon (Bardi), Helen Milroy (Palyku), Belle Selkirk (Noongar), Ashleigh Wright (Nunga), Kahli Regan (Wongi and Noongar), Shraddha Kashyap, Rama Agung‐Igusti, Joanna Alexi, Abigail Bray, Joan Chan, Ee Pin Chang, Sze Wing Georgiana Cheuk, Jemma Collova, Kate Derry, Chontel Gibson (Gamilaraay)

**Affiliations:** ^1^ Poche Centre for Indigenous Health University of Western Australia Perth WA; ^2^ Jumbunna Institute for Indigenous Education and Research University of Technology Sydney Sydney NSW; ^3^ Suicide Prevention Australia Sydney NSW; ^4^ Neuroscience Research Australia Sydney NSW; ^5^ The ARC Centre of Excellence for Indigenous Futures Brisbane QLD

**Keywords:** Cultural competency

## Author contributions

The authorship order for this article was led by and decided by Pat Dudgeon and Helen Milroy, based on work conducted within and reported by the team. Before that decision, each team member was provided an opportunity to write a summary of their contributions to the article, as well as recognise other authors’ roles and responsibilities in the process. The authors’ contributions, as per the CRediT for this article are as follows, noting that only the relevant roles from CRediT are included: Conceptualisation: Pat Dudgeon (PD) and Helen Milroy (HM) provided leadership for the development and application of Indigenous authorship principles, including holding space for conversations and activities to support the learning within the team. The remaining authors learn, practice and support the application of Indigenous authorship principles. Chontel Gibson (CG) and Shraddha Kashyap (SK) led the conceptualisation of the article in consultation with the remaining authors. Methodology: CG led the methodological approach to design and write the article. HM and PD provided leadership and advice. Supervision: CG and SK provided everyday supervision and guidance to draft the article. PD and HM provided strategic oversight. Project administration: CG and SK led the administration aspects of this article, including discussions and development. Writing – original draft: led by CG and SK with strategic oversight by PD and HM. Various support provided by the remaining authors. Writing – reviewing and editing: led by CG, Ashleigh Wright, Jemma Rose Collova and Rama Agung‐Igusti, with strategic advice, and review by PD and HM. Various support provided by the remaining authors.

## Positionality

Indigenous relationality is a shared principle that is practiced by Indigenous peoples across the world.^1^, ^2^ It requires people to share cultural connections, along with intentions. In this article, we illustrate our positionality by using a similar approach to Bullen and colleagues.^3^

### Acknowledgement to Country

We pay respects to the Elders and people of the Whadjuk Noongar nation, where our primary office is located. We acknowledge that the idea for this article originated on Yawuru Country and the manuscript was written on multiple Aboriginal Nations, including Gamilaraay, Yuin, Miriwoong and finally, Whadjuk and Wadandi Noongar. We extend our respect to Elders, Aboriginal and Torres Strait Islander peoples and nations across the continent now known by many as Australia.

### Introducing our teams and work

The authors of this article are a collective of Aboriginal and non‐Indigenous researchers who work together in two Aboriginal‐led research teams. Helen Milroy and Pat Dudgeon lead the Transforming Indigenous Mental Health and Wellbeing (TIMHWB) project, and the Centre of Best Practice in Aboriginal and Torres Strait Islander Suicide Prevention (CBPATSISP). Our teams promote Aboriginal and Torres Strait Islander self‐determination and strengthen Aboriginal and Torres Strait Islander peoples’ wellbeing. For example, we work in genuine partnership with Aboriginal Community Controlled Health Organisations through participatory approaches.^4^, ^5^, ^6^, ^7^ We strive to embed cultural safety into our research processes and co‐design methodologies.^8^, ^9^ We collaborate with universities across Australia to decolonise psychology curriculums.^10^

### Introducing and positioning authors

Members of both TIMHWB and CBPATSISP form the authorship of this article, and their positioning is detailed in the Box. We developed this article as a collective and as such, when we use pronouns such as “we” and “our,” we do so as a collective of both Aboriginal and non‐Aboriginal authors. We recognise that we are on decolonising journeys, as a collective and as individuals. We write in the spirit of collegiality and solidarity within our team and in alignment with others leading and/or actively participating in decolonial work.

Box 1 **Author position statements**

**Table:** 

Initials	Positioning
Pat Dudgeon	Professor Pat Dudgeon is a Bardi woman from the Kimberley. She is a psychologist and Fellow of the Australian Psychological Society and a researcher at the University of Western Australia's School of Indigenous Studies. Her area of research includes Indigenous social and emotional wellbeing and suicide prevention.
Helen Milroy	Professor Helen Milroy is a descendant of the Palyku people of the Pilbara region of Western Australia and she was born and educated in Perth. Australia's first Indigenous doctor and psychiatrist, Helen studied medicine at the University of Western Australia (UWA). She has contributed extensively to the field of Aboriginal and Torres Strait Islander peoples’ mental health and wellbeing, with a focus on children.
Belle Selkirk	Belle Selkirk is a cis‐gender Noongar woman and mother, privileged to live on Wadandi Boodja where she grew up. She is a clinical psychologist and research fellow in the Transforming Indigenous Mental Health and Wellbeing project focusing on indigenous psychology, decolonising psychology education and cultural safety in psychological practice.
Ashleigh Wright	Ash Wright is a Nunga woman raised Muruwari way. She is a provisional psychologist and PhD candidate whose research project supports collective self‐governance processes by Aboriginal communities to determine the “attachment” contexts or relationships of belonging in which Aboriginal children should be loved and cared for.
Kahli Regan	Kahli Regan is a Wongi and Noongar yorga (woman) raised on her nanna's country of the Goldfields before relocating to Wadjuk/Bingerup Noongar Boodja. Kahli is a PhD student and provisional psychologist whose research focuses on improving outcomes for Aboriginal young peoples and their families accessing mainstream mental health care.
Shraddha Kashyap	Dr Shraddha Kashyap has Indian heritage, was born and grew up in Kenya, and moved with her family to Boorloo, Whadjuk Noongar Boodja (Perth, WA) around 20 years ago. Shraddha uses she/her pronouns. She is an early career researcher and clinical psychologist. Shraddha's work focuses on how mental health services can be more culturally safe. As an immigrant living on Whadjuk Noongar Boodja, she prioritises her learnings to decolonise her way of working in the field of mental health to privilege Aboriginal and Torres Strait Islander worldviews and ways of knowing, being and doing.
Rama Agung‐Igusti	Dr Rama Agung‐Igusti is an early career researcher that lives and works on Whadjuk Noongar Boodja. He is descended from Anglo‐Celtic settlers and Austrian and Balinese migrants. Rama situates his research in the fields of community and Indigenous psychology. His work has involved documenting the dynamics of race in settler colonial Australia, and the ways racialised communities resist and respond to coloniality.
Joanna Alexi	Dr Joanna Alexi has Cypriot heritage, was born in Larrakia Country, and is now living on Whadjuk Noongar Boodja. Joanna is a research fellow in the Transforming Indigenous Mental Health and Wellbeing project, where her work focuses on decolonising psychology education and mental health care systems.
Abigail Bray	Dr Abigail Bray — she/her — born in Gwynedd (North Wales) and grew up in Kernow (Cornwall) with Celtic heritage, affiliated with Transforming Indigenous Mental Health and Wellbeing, UWA, and has practiced citational justice for a decade.
Joan Chan	Dr Joan Chan has Chinese heritage and moved to Boorloo, Whadjuk Noongar Boodja 10 years ago. Joan has worked to identify best practice Indigenous suicide prevention programs and services under the supervision of Professor Pat Dudgeon for about two years.
Ee Pin Chang	Dr Ee Pin Chang has Chinese heritage, moved to Whadjuk Noongar Boodja in 2010, and currently lives on Wurundjeri Country. Her research with the Centre of Best Practice in Aboriginal and Torres Strait Islander Suicide Prevention, UWA, focused on enhancing the social and emotional wellbeing (SEWB) of Aboriginal and Torres Strait Islander people in the criminal justice system. She continues to learn ways of working with the diverse Aboriginal and Torres Strait Islander people and academics to improve outcomes for Aboriginal and Torres Strait Islander people who are the traditional custodians of the lands that we all live and work on. Her current research aims to foster a healthy start to life for Aboriginal families.
Sze Wing Georgiana	Dr Georgiana Cheuk is from Hong Kong. She moved to Wadjuk Noongar Boodja to pursue study in psychology in 2010. She studies human language development and is currently working to promote suicide prevention for Aboriginal and Torres Strait Islander people. Her research focuses on human cognition and suicide prevention approaches for Aboriginal and Torres Strait Islander people.
Jemma Collova	Dr Jemma Collova is a cis‐gender woman whose grandparents migrated from Italy to Noongar Boodja in the 1950s. She is currently living on unceded Miriwoong Dawang with her husband. Jemma was educated under a Western paradigm of psychological science, and has spent the last few years continuing to decolonise her worldview and understandings of mental health and wellbeing. She is a researcher with the TIMHWB and CBPATSISP teams, and is motivated by work which aims to promote a more equitable and culturally safe mental health system.
Kate Derry	Dr Kate Derry, of Burmese immigrant and Irish/English settler heritage, was born and raised on unceded Wadjuk Boodja, where she lives with her husband and children. Her academic journey, shaped by a predominantly White and Western education in Australia, often felt disconnected from the cultural diversity she experienced growing up. Through her PhD on narcissism, she examined imbalances within Western culture, particularly capitalism, individualism, and the disconnection from community and mindfulness. Kate's work as a research fellow with TIMHWB has challenged her to decolonise her worldview and approach to research and reflection, enriching both her professional and personal development.
Chontel Gibson	Chontel is a Gamilaraay yinaar (woman), with family connections (from her mother and grandmother) to the Weatherall, Kennedy and Thorne families. Chontel is currently living on Wiradjuri Country. Chontel is able‐bodied, cis‐gender and uses she/her pronouns. While Chontel has participated in many Western education programs, she has done so under the guidance and support of Aboriginal and Torres Strait Islander networks, both professional and community‐based. Her professional work sits within the frame of decoloniality, with the aim of privileging Aboriginal and Torres Strait Islander peoples’ social and emotional wellbeing in health, education and policy.

### Conceptualisation of this article

The idea for this article originated in 2020. Our team was yarning about how to best acknowledge and privilege Indigenous peoples in research. This included mechanisms for Indigenous peoples to “maintain, control, protect and develop their cultural heritage, traditional knowledge and traditional cultural expressions, as well as the manifestations” of Indigenous Knowledges, such as sciences, health and wellbeing information.^11^ In more recent times, we used our teams’ Indigenous Research Methodologies Community of Practice to critically reflect on our principles and practices relating to Indigenous peoples’ rights within research processes, including Indigenous authorship.

### Rationale for article

Our work focuses on priorities identified by and/or with Aboriginal and Torres Strait Islander communities. Furthermore, our approaches privilege Aboriginal and Torres Strait Islander peoples’ ways of knowing, being and doing, in multiple ways, some of which we highlight below. The aim of this article is to build on existing literature, by sharing our principles and practices, in relation to respecting, recognising and honouring Indigenous Knowledges, including the application of Indigenous authorship principles. This article aims to elicit further critical reflexivity, including how both individuals and teams work with Aboriginal and Torres Strait Islander peoples, and Indigenous peoples globally. This article is designed for health research teams who work with Aboriginal and Torres Strait Islander people. However, the principles are also relevant to others who work and co‐design initiatives with Aboriginal and Torres Strait Islander peoples and indeed Indigenous peoples across the globe. Our principles and practices promote placed‐based and local knowledges, and as such, we do not apply or expect a one‐approach‐fits‐all communities and/or contexts. We provide examples of how our principles inform our practice. However, due to the highly nuanced and complex nature of such practices, it is beyond the scope of this article to share implementation strategies and challenges. The principles outlined in this article lay the foundation for future work, which should continue to critically engage with such challenges.

## Introduction

Aboriginal and Torres Strait Islander peoples’ knowledges and practices are grounded in the principles of relationality, which are foundational for flourishing communities.^3^ The historical and contemporary legacies of colonisation remain in everyday practices. These colonial legacies are a product of and result in racism, whereby there are deliberate attempts and/or everyday practices that destroy Indigenous Knowledges, such as languages, principles of relationality and cultural practices.^12^, ^13^ Western research paradigms, which are grounded in colonial ideology, have played a significant role in that destruction. Western researchers, the academy and disciplines produced within the academy, are contexts that position non‐Indigenous people, along with their processes and systems, as being the “knower” of Indigenous peoples, including Indigenous Knowledges. Within these same colonial research systems, Indigenous peoples are viewed as objects to be known, and Indigenous Knowledges as a commodity that is extracted, devalued and rearticulated through a Western lens, for the benefits of Western communities.^13^ Despite that context, Aboriginal and Torres Strait Islander peoples continue to resist, advocate and lead solutions that maintain, restore or rebuild Indigenous Knowledges.^13^, ^14^, ^15^, ^16^ Self‐determination and self‐governance are central to Indigenous peoples’ resistance, advocacy and leadership. They are also central for Indigenous protection, production, ownership and dissemination of Indigenous Knowledges.^1^, ^12^, ^13^, ^17^ One of many key examples where Indigenous peoples led the Indigenous rights movements, including self‐determination for Indigenous Knowledges and practices, was via the *United Nations Declaration on the Rights of Indigenous Peoples (UNDRIP)* and then the *Community Guide for the United Nations Declaration on the Rights of Indigenous Peoples* (Community Guide).^11^, ^18^ UNDRIP recognises and respects Indigenous peoples’ individual and collective rights to “maintain, control, protect, and develop” their knowledge systems along with “intellectual property over such cultural heritage, traditional knowledge, and traditional cultural expressions”.^11^ The rights asserted in the UNDRIP are fundamental and foundational in the process to transform colonial institutions, including the academy. The Community Guide is a comprehensive illustration of how the UNDRIP can be implemented with Aboriginal and Torres Strait Islander peoples.^18^ Epistemic justice, which is the recognition that Indigenous peoples (and all people) hold important knowledge, is central in any Indigenous rights‐based approaches.^19^ Indigenous human rights not only recognise that Indigenous peoples hold important knowledges but assert that Indigenous people are the rightful owners, who should maintain leadership, governance and connections with Indigenous Knowledges.^17^, ^18^

## Decolonisation

Colonisation stems from, as well as perpetuates racial imbalances of knowledge, knowledge production and knowledge practice. Decolonisation and decoloniality are a few of many tools used in attempts to dismantle, hinder, reverse, stop or remove colonising practices, with the aim of privileging the rights of Indigenous people.^13^, ^20^, ^21^, ^22^ We acknowledge the various, and sometimes conflicting conceptualisations and applications of decolonising and decolonial practices. These conflicts are influenced by place, people and socio‐political contexts, including the lack of transformative actions that should be of benefit to Indigenous peoples.^21^, ^23^, ^24^, ^25^, ^26^ Three key features, of the many, relating to decolonial and decolonising practices that we implement in our team are described below.

### Establish and understand positionality

Positionality is where one speaks from; it is reflective of values, beliefs and worldviews and how these underpin daily life.^1^ For Indigenous peoples, positionality is reliant on relationality, whereby relationships to Country, family and community underpin values, beliefs and worldviews.^1^, ^3^ Positioning includes one's professional context and intentions of research, as much as it is about positioning within the workplace.^21^ Furthermore, understanding one's workplace and the relationships formed with Indigenous communities, past and present, is essential. Positioning in context of colonisation is also important. Non‐Indigenous people need to understand their own positioning in relation to colonisation, including privileges associated with unearned power.^20^ Whereas Indigenous peoples' positioning with colonisation is linked with both historical and contemporary forms of oppression, which aims to eradicate Indigenous peoples and knowledges. Indigenous peoples have another link to colonisation; one that is associated with survival, resistance and a reclamation of Indigenous Knowledges and practices.

### Privileging Indigenous peoples

Honouring and sharing Indigenous peoples’ voices, including ways of knowing, being and doing, are paramount. There are multiple ways to value Indigenous peoples in research processes, such as using Indigenous research methodologies (eg, arts‐based methodologies, Aboriginal Participatory Action Research and yarning), recognising Indigenous peoples as knowledge holders within academic authorship processes, and upholding Indigenous leadership and governance in each step of the research process.^13^, ^20^, ^27^ The inclusion of Indigenous peoples as leaders in the research process demonstrates value of Indigenous peoples and a shift in power imbalances between the researcher and the researched. The denial of Indigenous leadership and other Indigenous ways of knowing, being and doing, could very much reflect a process embedded within or reflective of colonisation of Indigenous peoples.

### Transformative actions

While it is important to listen to Indigenous peoples, Moreton‐Robinson^16^ cautions non‐Indigenous peoples that “sitting with, listening to, hearing and remembering” Indigenous peoples voices can become a substitute for not addressing the ongoing colonial violence on Indigenous people.^16^ Hence the call for transformative actions, including transparent and accountable evaluation processes, is vital. Transformative actions are best done within and across institutions. For example, in psychology, the key national bodies that inform the socialisation and education of the profession now include policies and frameworks that promote and mandate cultural safety, decolonisation and decolonial practices (unpublished data). In mental health, the development of the Aboriginal and Torres Strait Islander social and emotional wellbeing (SEWB) model has created a framework that Aboriginal and Torres Strait Islander organisations and policy makers use to create and/or promote culturally safe health services (unpublished data). Important to note is that transformative actions may feel like a disruption to colonial institutions, such as universities and health facilities. However, these disruptions are nothing more than introducing justice‐based approaches, including to the relationships between Indigenous people and non‐Indigenous people. As such, this disruption should centre on an interrogation and a relinquishing of white racial privilege, so that Indigenous peoples’ self‐determination and governance is an everyday practice.^16^

## Indigenous health research

To engage with Indigenous health research, one must first understand that diverse Indigenous Knowledges exist. For this article, we view Indigenous Knowledges as including cultural knowledges (both past and present), Indigenous peoples’ lived experiences, and finally, new knowledges that may sometimes combine both Western and Indigenous Knowledges. We acknowledge the diversity of lived experiences and use a variety of innovative conceptual tools that are reflective of and built on Indigenous Knowledges, such as Milroy's Dance of Life, and Gee and colleagues’ SEWB model, which underpin our work.^28^, ^29^ Indigenous Knowledges are reflected in the holistic approaches to health, wellbeing and life. Holistic approaches encompass multiple knowledges relating to culture, spirituality, social and health, all of which are founded in Country and Lore — stories, sciences and practices.^30^, ^31^ Yunkaporta, as cited in Zubrzycki and colleagues^32^ provides a range of cultural protocols for engaging with Aboriginal and Torres Strait Islander knowledge. Examples of cultural protocols include using cultural processes, such as yarning, stories and art, to engage with Aboriginal and Torres Strait Islander knowledge; understanding one's positioning; settling one's own fears and discomfort, and finally, ensuring that any use of Aboriginal and Torres Strait Islander knowledges benefits Aboriginal and Torres Strait Islander communities.^32^

Cultural protocols also exist within research processes. Aboriginal Participatory Action Research (APAR) illustrates cultural protocols used within research relating to social and emotional wellbeing. APAR values Aboriginal and Torres Strait Islander peoples’ knowledges.^20^ It provides a decolonising and rights‐based Indigenous research methodology. The main elements of APAR centre on localised knowledge generation and Aboriginal and Torres Strait Islander peoples as co‐researchers; Indigenous peoples as experts‐by‐experience of their own health, families and communities; Indigenous leadership and governance, research translation and dissemination tailored by and for local communities; application of Indigenous ethical practices and finally, implementation of Swann and Raphael's nine guiding principles that underpin SEWB.^20^

Smith observes that the past two decades resulted in rapid growth of Indigenous research.^13^ Similarly, a SEWB literature review revealed a large volume of research that was led and governed by Aboriginal and Torres Strait Islander peoples (unpublished data). The expansion of Indigenous research illustrates how Indigenous peoples’ knowledges and practices can transcend and enact Indigenous human rights within the academy. Despite Indigenous Knowledges transcending the academy, Smith maintains that Western disciplines and institutions continue to have difficulty acknowledging the contributions of Indigenous Knowledges.^13^ The lack of acknowledgement can be demonstrated in Western disciplines and researchers’ patterns of citation that often exclude or undervalue racialised academics.^33^ The lack of Indigenous Knowledges can also be demonstrated in challenges such as the limited amount of Indigenous content taught in education programs; the limited numbers of identified Indigenous positions in the academy and limited access to culturally safe institutions (eg, university programs and health services), which deters Indigenous peoples’ use of the institutions, as consumers, staff, students etc.^8^, ^21^, ^34^, ^35^ These problems result from and in, what Emery‐Whittington refers to as the ongoing transmission of colonial oppression.^36^ We acknowledge that efforts have been made to mitigate transmissions of colonial oppression.^21^ For example, while Aboriginal and Torres Strait Islander social and emotional wellbeing has been implemented by many Aboriginal community‐controlled organisations, the uptake in mainstream health organisations is not as apparent (unpublished data).

## Indigenous authorship

Indigenous peoples are the rightful owners of Indigenous Knowledges, and in the research process, Indigenous authorship is one mechanism to enact that right. Indigenous publishers have long advocated for Indigenous authorship. For example, the *Guidelines for the ethical publishing of Aboriginal and Torres Strait Islander authors and research from those communities* illustrate the problems with copyright laws in Australia, in that they grant ownership to the person who writes down the Indigenous Knowledge, rather than the rightful owner of the Indigenous Knowledge.^37^ Janke^38^ reinforces how copyright laws and intellectual property rules privilege non‐Indigenous people, who often benefit from the laws and rules, especially when it relates to Indigenous peoples. Journals are becoming increasingly aware of their responsibilities to promote Indigenous authorship in the publication process.^39^, ^40^

Scholarly articles are now revealing guidelines, tools and checklists to support the process of Indigenous authorship. For example, Kennedy and colleagues illustrate the importance of Indigenous authorship, emphasising the merit of positioning Indigenous authors as first and last in the authorship order, when using an Indigenous method/methodology.^41^ CAVAL and the Indigenous Archives Collective guidelines, along with its Indigenous Knowledge Attribution Toolkit, were designed for undergraduate students.^42^ These resources aim to support students to choose references that are strength‐based and inclusive of Indigenous leadership, including Indigenous authorship.^42^ CAVAL and the Indigenous Archives Collective also provide a process for acknowledging authors’ cultural connections within texts of publications and in the reference list.^42^

The *Australia state of environment report* released a guideline and strategy that committed to Indigenous co‐authorship, reflecting the importance of and inclusion of Indigenous Knowledges.^43^ Finally, Indigenous data sovereignty has grown momentum in the past five years, and rightly so, given that Indigenous data, and therefore Indigenous Knowledges, are best left in the hands of Indigenous peoples who are connected to that knowledge, such as local communities and professional communities.^17^ Indigenous data sovereignty principles, to some extent, are embedded in the new Framework for Governance of Indigenous Data.^44^, ^45^ Indeed, all previously mentioned frameworks provide examples on how to enact and/or assert UNDRIP's Article 31 in relation to Indigenous peoples having “the right to maintain, control, protect and develop their intellectual property over such cultural heritage, traditional knowledge, and traditional cultural expressions”.^11^

## Sharing our teams’ principles and practices to guide Indigenous authorship

Our principles in relation to sharing Indigenous Knowledges and centring Indigenous authorship have long been practised. Although our principles remain constant over time and within projects, our practices tend to evolve and/or change to reflect cultural protocols. For this article, we refined our principles and provided an example of how we practise each principle. We encourage readers to honour place‐based knowledge, and this will involve working with Aboriginal and Torres Strait Islander people who are key stakeholders in research, to understand the best principles and actions relevant to each specific context. Our principles and practices are as follows.

### Principle 1: Do no harm

Our team applies the principle of do no harm to Indigenous authorship. Core to this principle is that authors accept they have a responsibility to do no harm to Aboriginal and Torres Strait Islander peoples, Country and cultural connections.^45^, ^46^, ^47^ That responsibility means authors are accountable to follow Aboriginal and Torres Strait Islander peoples’ cultural protocols, critically reflecting on one's positionality and privileging Indigenous ways of knowing, being and doing.^45^, ^46^, ^47^ Doing no harm is not one act, but a complex, iterative and holistic approach that involves practicing the remaining principles.

### Principle 2: Privilege Indigenous ways of knowing, being and doing

We recognise the multiple ways in which Aboriginal and Torres Strait Islander peoples know, and therefore, there are multiple ways of being and doing.^46^, ^48^ For example, we take time to understand and apply cultural protocols relating to the sharing of Indigenous Knowledges in families and communities. Older people, Elders, Knowledge Holders and Traditional Landowners hold unique positions in sharing knowledges, such as knowledges relating to culture, spirituality, Lore, history and more.^15^, ^49^ As such, significant community members are active leaders in our team research processes, including providing crucial leadership during relationship building, data analysis, Indigenous governance, and critically reviewing articles before publication. Given these roles and responsibilities, it is imperative that Aboriginal and Torres Strait Islander key community stakeholders take their rightful place as authors in publications. The inclusion of Aboriginal and Torres Strait Islander peoples’ ways of knowing, being and doing and as authors increases the benefits for and with communities. For example, for Indigenous Knowledges to exist in an authentic and meaningful manner, they need to exist in relation to Indigenous people and Country of where the knowledge/s originated and/or was shared.^50^, ^51^ Therefore, the inclusion of Aboriginal and Torres Strait Islander authors is likely to increase trust in the research process, and subsequently in the research translation in communities.

### Principle 3: Develop and maintain respectful processes of engagement

Meaningful engagement with Aboriginal and Torres Strait Islander peoples is based on trust, maintaining ongoing rapport and relationships.^46^, ^48^ The engagement process involves listening, observing, sharing, using strength‐based discourse, checking in and responding in respectful ways to Indigenous Knowledges shared. When working with Aboriginal and Torres Strait Islander peoples in research, trust must be earned and not assumed. Building trust and rapport, along with following the principles of relationality and reciprocity are important aspects of research valued by Aboriginal peoples.^52^ For example, our team embeds flexible and empowering processes that accommodate for the research direction to change in response to what Aboriginal and Torres Strait Islander peoples tell us. Our relationships with Aboriginal and Torres Strait Islander peoples often extend beyond the typical research processes, whereby we take time to build authentic connections that last beyond the life of the research, rather than viewing participants as the “subjects” of research.

### Principle 4: Actively encourage and promote community ownership and control

We acknowledge and embrace Indigenous Knowledges and value Indigenous peoples who share individual or community knowledges, and we do this by honouring relevant cultural protocols for sharing Indigenous Knowledges.^46^, ^48^ Our research processes include Aboriginal and Torres Strait Islander peoples in the research team and in governance structures, who are reflective of communities involved in the research. Aboriginal and Torres Strait Islander leadership ensures community ownership of the research process and outcomes, including and not limited to decisions relating to Indigenous data sovereignty and Indigenous authorship. For example, similarly to Trudgett and colleagues’ definition of ownership, we assert Aboriginal and Torres Strait Islander peoples’ right to make decisions about the cultural protocols, methods and processes within the research process^17^ as well as how research outcomes are disseminated. We deeply consider how Indigenous authorship will be honoured on all resources disseminated.

### Principle 5: Honour the diverse identities, roles and contributions of Aboriginal and Torres Strait Islander peoples

We acknowledge the unique contributions of Aboriginal and Torres Strait Islander peoples.^46^, ^48^ We recognise that research cannot take place without Aboriginal and Torres Strait Islander peoples’ guidance, support, input, leadership and ongoing involvement. In our research, we assert the rights of Aboriginal and Torres Strait Islander peoples to determine the responsibilities of individuals in that community and what those responsibilities are to their community.^18^ That means Aboriginal and Torres Strait Islander peoples are viewed as more than just the subject of research projects, but they are respected in their multiple roles and responsibilities, including being “knowers” of knowledge, especially their own knowledges.^53^ For example, Aboriginal and Torres Strait Islander peoples are knowledge holders, as in the roles of Elders, Traditional Owners, and/or the expert of their own lived experience. Aboriginal and Torres Strait Islander peoples also hold roles in the research process, such as being researchers, supervisors, community consultants and members of governance groups. We allow time and space for Aboriginal and Torres Strait Islander peoples to fulfil diverse identities, roles and responsibilities. Sometimes, allowing time and space means research is not the highest priority, and other business, such as sorry business (ceremonies relating to bereavement) or family business take precedent.

### Principle 6: Enact a process for informed and ongoing consent

We recognise the necessity of enacting a process for free, prior, informed and ongoing individual and community consent, when considering Aboriginal and Torres Strait Islander peoples’ engagement in research, including when determining authorship.^46^, ^48^, ^54^ We understand that how we design, engage and conduct research with Indigenous peoples enables the exercise of self‐determination, including Indigenous community decision‐making processes and Indigenous peoples’ right to choose how they want to live.^11^, ^46^, ^48^ Our team is responsible for ensuring all parties remain informed throughout the research journey and that information is conveyed in a way that has meaning, context and value for the person receiving information.

### Principle 7: Facilitate dialogues for Aboriginal and Torres Strait Islander peoples to determine the benefits

We ensure that Aboriginal and Torres Strait Islander peoples are fully informed, and are aware of any benefits of the research, including the benefits of being an author.^46^, ^48^ What constitutes a benefit will vary from person to person, and community to community. For example, an Elder in the community may sometimes wish to impart their knowledge to the broader community via a research publication. We acknowledge that Aboriginal and Torres Strait Islander peoples will have different opinions on authorship, including preferences for authorship order. These preferences may change depending on each specific research context, and the relationality of each person within that context. Sometimes, there is a benefit for Aboriginal and Torres Strait Islander peoples to not be an author. For example, Aboriginal and Torres Strait Islander peoples participating in the research may not wish to be identified or may not value being an author on an academic paper. However, when this occurs, we ensure that a respectful and considerate process is followed, relationships maintained and critical dialogue for the same occurs. The benefits of engaging in a dialogue about authorship and determining the most appropriate authorship team (and order) will last beyond the life of the project. For example, that dialogue and inclusion of Indigenous authorship, if done in an authentic manner, is an act of decoloniality, whereby Indigenous people become the “knowers” and not just the “subject” in the research.

### Principle 8: Critically engage in transformative actions that mitigate transmission of colonial oppression

We acknowledge the ongoing nature of colonisation, including the ongoing racial power imbalances, which are embedded in colonial institutions’ systems, structures and everyday practices.^46^, ^48^ That everyday practice includes what Bargallie and colleagues^55^ state as being the “tendency to dissociate race from invasion and colonisation and to speak of colonialism as a ‘legacy’”, which is perhaps one reason behind Indigenous rights not being recognised or taken seriously. With that context in mind, we critically reflect on the colonial epistemic violence to ensure that our ways of working continue to value Aboriginal and Torres Strait Islander peoples. For example, we appreciate that universities prioritise academics’ workloads to centre on publications and less on other forms of research dissemination. However, we continue to work with communities to ensure that research processes and outcomes are of value to communities, which means extending beyond the usual practices for dissemination. While advocacy occurs across the university sector, there is an abundance of opportunities for further systemic and practice changes.

### Principle 9: De‐centre oneself and work as a collective

We recognise the importance of de‐centring oneself and our team from colonial practices. We work collectively, which aligns with Aboriginal and Torres Strait Islander peoples’ concepts of selfhood within collectivist cultures.^20^, ^46^, ^48^ Our research is motivated by the aims of reclaiming, reconstituting and restoring Indigenous Knowledges, which inevitably relates to positively influencing SEWB.^33^ These processes are embedded in Aboriginal and Torres Strait Islander peoples’ self‐determination, and as such Indigenous people leading the research process and outcome. We take the time to build relationships and when necessary, we advocate for systemic changes and when that is not successful, then we negotiate procedural changes, so that we can still uphold Aboriginal and Torres Strait Islander peoples’ ways of knowing, being and doing.

## Conclusion

Indigenous peoples, both in and out of the academy are advocating and leading Indigenous rights initiatives. As a result, Indigenous researchers, Indigenous research and Indigenous Knowledges are on the incline in the academy. Despite that rise, epistemic injustices are still apparent in the academy, including the health and health‐related disciplines’ knowledge and practices, which are produced within the academy. Our team has developed nine principles to guide Indigenous authorship, and these principles are underpinned by and support Indigenous peoples’ rights, including epistemic justice, specifically as it relates to Indigenous authorship. We appreciate that our perspective article shares some of our practices, which have and will continue to evolve over time. We encourage other research teams who work with Indigenous people and knowledges, to share their principles and practices relating to epistemic justice. We appreciate that epistemic justice is a relatively new practice for non‐Indigenous researchers, and as such, making the time and taking the lead from Indigenous key stakeholders about principles, practices and implementation strategies are necessary.

## Competing interests

No relevant disclosures.

## Provenance

Not commissioned; externally peer reviewed.
